# High-Temperature Induced Changes of Extracellular Metabolites in *Pleurotus ostreatus* and Their Positive Effects on the Growth of *Trichoderma asperellum*

**DOI:** 10.3389/fmicb.2018.00010

**Published:** 2018-01-19

**Authors:** Zhiheng Qiu, Xiangli Wu, Jinxia Zhang, Chenyang Huang

**Affiliations:** ^1^Institute of Agricultural Resources and Regional Planning, Chinese Academy of Agricultural Sciences, Beijing, China; ^2^Key Laboratory of Microbial Resources, Ministry of Agriculture, Beijing, China

**Keywords:** *Pleurotus ostreatus*, green mold disease, high temperature, metabolites, *Trichoderma asperellum*, growth

## Abstract

*Pleurotus ostreatus* is a widely cultivated edible fungus in China. Green mold disease of *P. ostreatus* which can seriously affect yield is a common disease during cultivation. It occurs mostly after *P. ostreatus* mycelia have been subjected to high temperatures. However, little information is available on the relationship between high temperature and green mold disease. The aim of this study is to prove that extracellular metabolites of *P. ostreatus* affected by high temperature can promote the growth of *Trichoderma asperellum*. After *P. ostreatus* mycelia was subjected to high temperature, the extracellular fluid of *P. ostreatus* showed a higher promoting effect on mycelial growth and conidial germination of *T. asperellum*. The thiobarbituric acid reactive substance (TBARS) content reached the maximum after 48 h at 36°C. A comprehensive metabolite profiling strategy involving gas chromatography-mass spectrometry (GC/MS) combined with liquid chromatography-mass spectrometry (LC/MS) was used to analyze the changes of extracellular metabolites in response to high temperature. A total of 141 differential metabolites were identified, including 84.4% up-regulated and 15.6% down-regulated. Exogenous metabolites whose concentrations were increased after high temperature were randomly selected, and nearly all of them were able to promote the mycelial growth and conidial germination of *T. asperellum*. The combination of all selected exogenous metabolites also has the promotion effects on the mycelial growth and conidial germination of *T. asperellum* in a given concentration range *in vitro*. Overall, these results provide a first view that high temperature affects the extracellular metabolites of *P. ostreatus*, and the extensive change in metabolites promotes *T. asperellum* growth.

## Introduction

*Pleurotus ostreatus* known as the oyster mushroom is an important commercial mushroom which is widely cultivated all year round in China. Green mold disease is a common disease of *P. ostreatus*. Several cases of green mold disease have been found during the cultivation of *P. ostreatus* in many countries (Komon-Zelazowska et al., 2007; Blaszczyk et al., 2013). The disease can seriously reduce the growth and yield of oyster mushroom and lead to huge economic losses (Kredics et al., 2009). In past years, most reports have mainly focused on the infection mechanism of *Trichoderma* and the inhibition effect of *Trichoderma* spp. on the mycelial growth of other fungi (Amin et al., 2010; Eslaminejad Parizi et al., 2012; Shamoli et al., 2013). Our previous study has found that high temperature plays an important role in the cause of green mold disease (Qiu et al., 2017). However, extracellular metabolites of *P. ostreatus* especially after heat stress and their effects on the growth of *Trichoderma* have not been reported yet.

Green mold disease occurs in the cultivation of many kinds of mushrooms, and many studies have identified *Trichoderma* spp. associated with this disease (Park et al., 2006; Hatvani et al., 2007). In a biological control study, root exudates provided nutrients for the growth of *Trichoderma* spp. and facilitated its colonization at the roots (Vargas et al., 2009). In the process of *Trichoderma* colonization, *Trichoderma* conidia can quickly germinate into mycelium by using the nutrients in substrates. Due to the rapid growth rate, *Trichoderma* mycelium can also quickly occupy nutrient sites and space, utilize nutrients, and then degrade other fungal mycelia (Benítez et al., 2004). High temperature is a type of abiotic stress, which can activate multiple metabolic pathways in cells and affect mycelial growth and fruiting body differentiation during mushroom cultivation (Chang and Miles, 2004). Heat stress can cause an increase in intracellular production of reactive oxygen species (ROS) and lead to metabolic disorders (Mittler, 2002). In addition, high temperature can also cause membrane lipid peroxidation and increase the fluidity and permeability of the cell membrane (Apel and Hirt, 2004). As a result, more intracellular metabolites are able to leak from cells. Therefore, it is of positive significance to investigate the relationship between extracellular metabolites of *P. ostreatus* affected by high temperature and the outbreak of green mold disease.

Metabolomics is an ideal tool to analyze metabolite changes in response to environmental changes (Link et al., 2015). An effective method for extracting metabolites is a prerequisite for assessing the metabolite changes which is conductive to study the changes in intracellular metabolic pathways (Doerfler et al., 2013). Previously, some extraction methods have been employed for metabolites extraction, such as hot water, boiling ethanol, freezing-thawing in methanol, chloroform-methanol, etc. (de Koning and van Dam, 1992; Hajjaj et al., 1998; Maharjan and Ferenci, 2003; Bogialli et al., 2005). Currently, the chloroform-methanol (CM) method is a more acceptable method for metabolite extraction (Canelas et al., 2009).

Gas chromatography-mass spectrometry (GC/MS) is a highly efficient technology for identifying and profiling primary metabolites, such as organic acids, amino acids, sugars and some nonpolar compounds (Fiehn, 2008). However, it is difficult to detect nonvolatile, non-derivatized metabolites and some other molecules with this method. Liquid chromatography-mass spectrometry (LC/MS) can be used for determination of volatile compounds, polar compounds, thermally unstable compounds and high molecular weight compounds (Zhang et al., 2014; Xu et al., 2015). GC/MS and LC/MS complement each other, and a combination of these methods allows more comprehensive analysis of different compounds (Lee et al., 2013; Yao et al., 2013). The combination of the two methods have also been widely used to detect metabolites in mushrooms, such as *P. ostreatus* (Zhang et al., 2008), *Agaricus bisporus* (O'Gorman et al., 2011), and *Lentinula edodes* (Mata et al., 2014).

In order to fully understand the changes of the metabolites in the extracellular fluid of *P. ostreatus* before and after high temperature, a metabolomic approach was applied (i.e., GC/MS and LC/MS). In addition, the effects of exogenous metabolites of *P. ostreatus* on *T. asperellum* mycelial growth and conidial germination were investigated. The objective of this study was to analyze the effect of high temperature on extracellular metabolites production of *P. ostreatus* and test the effect of these metabolites on *T. asperellum* mycelial growth and conidial germination. Finding the cause of the outbreak of green mold disease will help us find a way to control it in future.

## Materials and methods

### Fungal strains, growth conditions, and collection of the samples

*Pleurotus ostreatus* P89 (CCMSSC 00389) was obtained from the China Center for Mushroom Spawn Standards and Control. *T. asperellum* T11 (ACCC 32725) was provided by Agricultural Culture Collection of China. For pure culture of *P. ostreatus*, samples of mycelial discs (5 mm) were cultured on potato dextrose agar at 28°C (PDA; Difco-Becton Dickinson, Sparks, MD). For collection of extracellular fluid of *P. ostreatus* mycelia, 10 pieces of *P. ostreatus* mycelial discs (5 mm) were inoculated into 250 mL Erlenmeyer flasks containing 100 mL of liquid minimal synthetic medium(MSM). One liter of MSM contained: 5 g glucose, 0.2 g MgSO_4_•7H_2_O, 0.9 g K_2_HPO_4_, 0.2 g KCl, 1.0 g NH_4_NO_3_, 0.002 g FeSO_4_•7H_2_O, 0.002 g MnSO_4_, 0.002 g ZnSO_4_ and distilled water. The flasks were incubated in rotary shaker (ZQLY-180F, Shanghai Zhichu Instrument Company Limited, Shanghai, China) at 150 rpm and 28°C for 10 days, and then transferred into incubators with different temperatures (28°, 32°, 36°, and 40°C) for 2 days. The extracellular liquid medium was collected by centrifuging (Sigma 3K30, Osterode, German), and the supernatant was filtered (Millex-GP, 0.22 μm, Merck Millipore Ltd).

### Effects of extracellular culture fluid of *P. ostreatus* on mycelial growth and conidial germination of *T. asperellum*

To detect the effects of extracellular fluid of *P. ostreatus* on mycelial growth and conidial germination of *T. asperellum*, 100 mL of extracellular fluid of *P. ostreatus* which had been treated with different temperatures (28°, 32°, 36°, and 40°C) for 2 days were mixed with 100 mL of 4% water agar. Then, T11 mycelial discs were placed in the center of the plates and incubated at 28°C for 3 days. Finally, the colony diameters were recorded. At the same time, T11 conidia were collected and diluted to the appropriate concentration (1.9 × 10^3^ CFU/mL). Then, 100 μL of conidial suspension were spread onto 2% water agar petri dishes containing different extracellular fluid, and then incubated at 28°C. The number of germinated conidia was recorded after 2 days of incubation. Every treatment group consisted of three replicates.

### Determination of TBARS content in *P. ostreatus* mycelia after high temperature treatment

The TBARS content is a reflection of oxidative damage to cell membrane lipid (Kong W. et al., 2012). To investigate the effect of high temperature on membrane lipid peroxidation, *P. ostreatus* P89 mycelial discs were placed in the center of sterile cellophane laying on PDA plates and incubated at 28°C for 5 days. Then, the plates were respectively placed at 28° and 36°C for various times (6, 12, 24, and 48 h). P89 mycelia were collected and stored at −80°C until analysis. TBARS was detected as described before with some modifications (Kong W. W. et al., 2012). Firstly, 0.2 g of P89 mycelia were ground into powder with liquid nitrogen, and then transferred into a 1.5 mL Eppendorf tube. Briefly, 0.5 mL of 5 % TCA was added. Then the mixture was extracted for 10 min in ice water bath. The supernatants were collected by centrifuging at 10,000 × g for 10 min and mixed with 0.5 mL of 0.67% TBA in a new Eppendorf tube. The mixture was subsequently incubated at 95°C for 30 min, then centrifuged at 10,000 × g for 10 min. The absorbance of the supernatant was measured at 532 nm and 600 nm wavelength using a UV-spectrophotometer (TU-1810, PERSEE, Beijing, China). All tests were performed in triplicate.

### Chemicals

All chemicals and solvents were of analytical or HPLC grade. Water, methanol (HPLC grade), acetonitrile (UHPLC grade), pyridine, n-hexane, methoxylamine hydrochloride (97%), *N*-methyl-*N*-trimethylsilyltrifluoroacetamid (MSTFA) with 1% trimethylchlorosilane (TMCS) were purchased from CNW Technologies GmbH (Düsseldorf, Germany). Trichloromethane was from Shanghai Chemical Reagent Co., Ltd. (Shanghai, China). 2-chloro-l-phenylalanine was purchased from Shanghai Hengchuang Bio-technology Co., Ltd. (Shanghai, China).

### Sample collection and preparation for metabolites measurements

Ten pieces of P89 mycelial discs (5 mm) were inoculated into 250 mL Erlenmeyer flasks containing 100 mL of MSM and incubated in rotary shaker at 150 rpm and 28°C for 10 days. Subsequently, flasks were transferred into rotary shaker with different temperatures (28° and 36°C) for 2 days. The extracellular fluid was collected by centrifugation and the supernatant was filtered with sterile syringe filters (0.22 μm). Finally, the culture filtrate was freeze-dried, and then ground into fine powder. Each set contains eight parallel samples.

For GC/MS analysis, to accurately determine extracellular metabolites levels of *P. ostreatus*, a chloroform-methanol (CM) method was used for metabolites extraction with some modification (Canelas et al., 2009). Firstly, 60 mg of sample was added into 4 mL glass vial and mixed with 20 μL of 2-chloro-l-phenylalanine (0.3 mg/mL) which was dissolved in methanol as the internal standard. Then, 2 mL of methanol: water (4:1, volume ratio) were added to every glass vial. An aliquot of 400 μL of chloroform was added to the samples, and samples were dispersed with pipette. Then, the sample was broken up by ultrasonic homogenizer (Fisher Scientific™ Model 120 Sonic Dismembrator, Ottawa, Canada) at 500 w for 6 min. Then, 800 μL of each sample was subsequently transferred into a new 1.5 mL Eppendorf tubes. After treating with ultrasonication for 20 min in ice water bath, the supernatant (500 μL) was collected by centrifugation (12,000 rpm, 4°C for 10 min), and then transferred into a glass vial and dried in a vacuum freeze dryer (Christ ALPHA 1-2 LD plus, Osterode, Germany). The sample was homogenized with 80 μL of methoxyamine hydrochloride (15 mg/mL in pyridine) using a vortex mixer. Subsequently, 80 μL of MSTFA with 1% TMCS were mixed the mixture after it was incubated at 37°C for 90 min. Briefly, 20 μL of n-hexane was added, and the solution was derivatized at 70°C for 60 min. Finally, the solution was placed at room temperature for 30 min before GC/MS analysis.

For LC/MS analysis, 60 mg sample was added to a glass vial and mixed with 4 mL of ice-cold 80 % (v/v) methanol and water (4:1, volume ratio). Subsequently, 400 μL of trichloromethane were added with 20 μL of internal standard (0.3 mg 2-chloro-l-phenylalanine/mL methanol). Then, the mixtures were homogenized using a vortex for 1 min and broken up by ultrasonic homogenizer for 6 min at 500 w in ice water bath. Then, 800 μL of homogenate were transferred into a 1.5 mL Eppendorf tube, and further ultrasonicated at room temperature for 20 min, and centrifuged at 15,000 rpm, 4°C for 10 min. Finally, 500 μL of supernatant was filtered (Millex-GP, 0.22 μm, Merck Millipore Ltd) before LC/MS analysis.

### GC/MS analysis

The derivatized samples were analyzed using an Agilent 7890B gas chromatography system coupled to an Agilent 5977A MSD system (Agilent, CA). The column was a DB-5MS fused-silica capillary (30 m × 0.25 mm × 0.25 μm, Agilent J & W Scientific, Folsom, CA, USA). Ultra- pure helium was used as the carrier gas at a flow of 1 mL/min. The inlet temperature was kept at 260°C. Injection volume was 1 μL with a 30:1 split ratio. The initial oven temperature was 90°C, then ramped to 180°C at a rate of 10°C/min, then to 240°C at 5°C/min, to 290°C at 25°C/min, and finally held at 290°C for 11 min. MS was used in electron impact mode (70 eV) and MS quadrupole was set to 150°C. Full-scan mode (m/z 50–450) was used to acquire MS data, and the solvent delay time was set to 5 min.

### LC/MS analysis

An aliquot of 500 μL sample was analyzed using an Agilent 1290 Infinity UHPLC coupled to an Agilent 6538 UHD and Accurate-Mass Q-TOF/MS system. Chromatographic separation was performed with an ACQUITY UPLC@HSS T3 chromatographic column (2.1 μm, 100 × 2.1 mm). The mobile phase included A (0.1% formic acid) and B [acetonitrile (containing 0.1% formic acid)]. The flow rate was 0.4 mL/min. The column was maintained at 45°C and separation was achieved using the following gradient: 5–80% B over 0–10 min, 80–100% B over 10–12.5 min, 100–5% B over 12.5–12.6 min, and 12.6–14 min holding at 5% B. Injection volume was 3 μL.

### Data analysis

The acquired MS data from GC/MS were analyzed by ChromaTOF software (v 4.34, LECO, St Joseph, MI). Metabolites were identified by the Fiehn database, which was linked to the ChromaTOF software. Briefly, statistic compare component was used to process the raw data. Sample information, peaks' name, mass/retention time and peak intensities were exported as the CSV file for multivariate statistical analysis. The internal standard was used for data quality control (reproducibility).

The raw data from LC/MS were converted into common format by Agilent MassHunter Qualitative software. Briefly, after alignment with Statistic Compare component by XCMS (https://xcmsonline.scripps.edu/). Three-dimension (3 D) data sets including sample name, mass/retention time and peak intensities were also applied. The internal standard was used for data quality control (reproducibility).

Principal component analysis (PCA) and orthogonal partial least-squares discriminant analysis (OPLS-DA) were performed to analyze the resulting data of GC/MS and LC/MS which were imported into a SIMCA (version 14.0, Umetrics, Umeå, Sweden), (Worley and Powers, 2016). The ellipse in score plots of the models which means the 95% confidence interval of the modeled variation is the Hotelling's T2 region. The R^2^X or R^2^Y and Q^2^ values are performed to evaluate the quality of the models. R^2^X or R^2^Y which indicates goodness of fit is the proportion of variance in the data explained by the models. Q^2^ which indicates predictability is the proportion of variance in the data predicted by the model, calculated by a cross-validation procedure. In order to determine the optimal number of principal components and avoid model over fitting, a default seven-round cross-validation in SIMCA was applied. Moreover, a permutation analysis (200 times) was also performed to validate the OPLS-DA models.

### Mycelial growth and conidial germination of *T. asperellum* under exogenous metabolites treatments

According to the GC/MS and LC/MS differential metabolites data, 3 metabolites were randomly selected from each category of metabolites whose content was increased after 36°C to analyze their effects on the growth and conidial germination of *T. asperellum*. Different concentration (0, 50, 100, and 250 μM) of exogenous metabolites (purchased from Sigma) were applied. T11 mycelial discs (5 mm) were inoculated in the center of 2% agar petri dishes containing different concentration of exogenous metabolites. Then, *T. asperellum* mycelial growth rate was calculated according to the colony diameters after incubation at 28°C. One hundred microliter of *T. asperellum* conidial suspension (1.23 × 10^3^ CFU/mL) were spread onto 2% water agar petri dishes containing different concentrations of exogenous metabolites. The number of germinated conidia was recorded 2 days after incubation at 28°C. Every treatment group was performed in triplicate. In addition, the combination of all selected metabolites at various concentrations (0, 50, 100, and 250 μM) were mixed with 4% water agar to detect their effects on mycelial growth, and were also mixed with 4% water agar to detect their effects on conidial germination of T11.

### Statistical data analysis

The differential metabolites for GC/MS and LC/MS were selected according to the combination of variable important in the projection (VIP) values threshold and *p*-values which derived from the OPLS-DA model and two-tailed Student's *t*-test on the normalized peak areas, respectively. Other data are presented as the mean standard deviation (SD). Analysis of variance (ANOVA) and Duncan's multiple range tests (*p* ≤ 0.05) were used to determine the significance. Error bars represent the standard deviation of three replicates.

## Results

### Extracellular fluid of *P. ostreatus* promoted mycelial growth and conidial germination of *T. asperellum*

**Figure 1:**
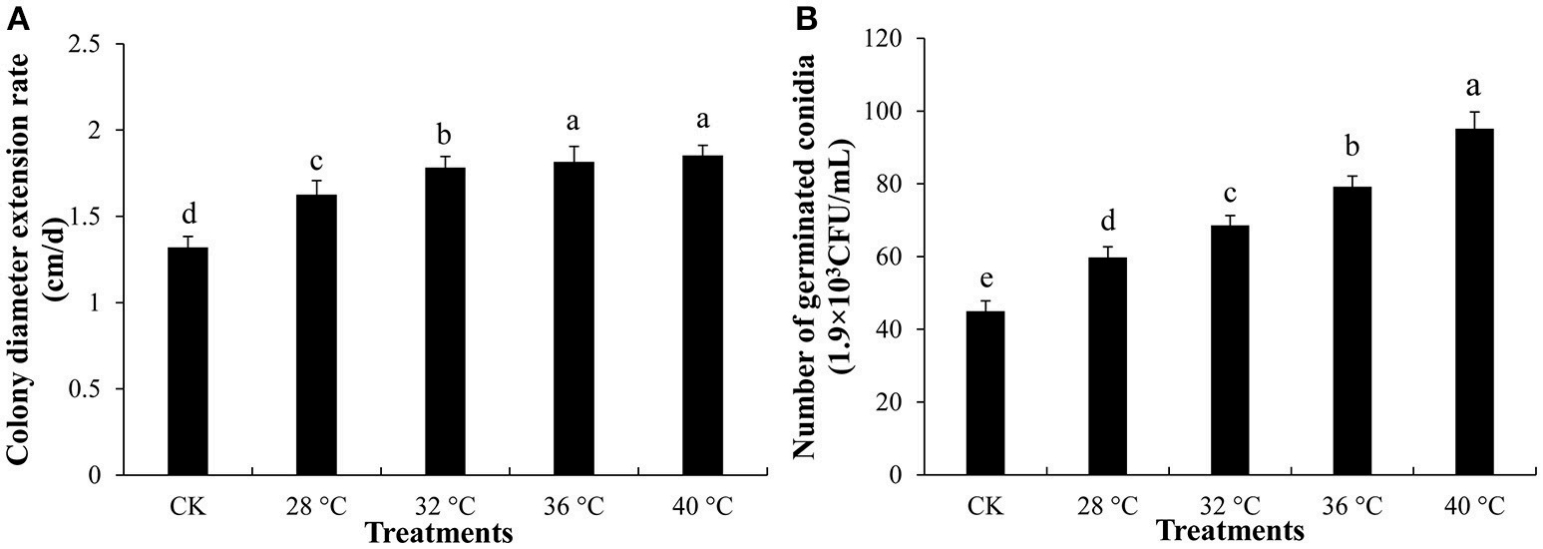
Mycelial growth and conidial germination of *T. asperellum*. **(A)** Mycelial growth on plates with extracellular fluid collected from different temperature treatment on. **(B)** Number of germinated conidia on plates with extracellular fluid which was treated with different temperature. CK was that 100 mL 4% water agar mixed with 100 mL sterile water. Data were analyzed by Duncan's ANOVA test. Error bars represent the standard deviation of three replicates. Different letters indicate significant differences between the columns (*P* ≤ 0.05 according to Duncan's multiple range test).

*Trichoderma asperellum* T11 mycelial growth and conidial germination were significantly more stimulated by extracellular fluid of *P. ostreatus* P89 (Figures 1A,B). T11 mycelia had a significantly (*p* ≤ 0.05) faster growth rate on plates mixed with extracellular fluid which had been treated with higher temperature (Figure 1A). The promoting effects of extracellular fluid obtained at 36° and 40°C were the highest, and T11 mycelial growth rates (1.82 and 1.85 cm/d, respectively) were significantly higher than those at 28° or 32°C. The extracellular fluid obtained at high temperatures also had promotional effect on the conidial germination (Figure 1B). The conidia had significantly (*p* ≤ 0.05) higher germination rate on plates with extracellular fluid collected from high temperature treated medium. The conidial germination rate was the lowest on plate with extracellular fluid collected from 28°C. In comparison with that collected from 28°C, conidial germination rate on plates with extracellular fluid collected from the treatment of 36° and 40°C were 32.4 and 59.2% higher, respectively. The extracellular fluid treated with high temperature exhibited similar stimulation effects on the mycelial growth and conidial germination.

### Effect of high temperature on membrane lipid peroxidation of *P. ostreatus* mycelia

**Figure 2:**
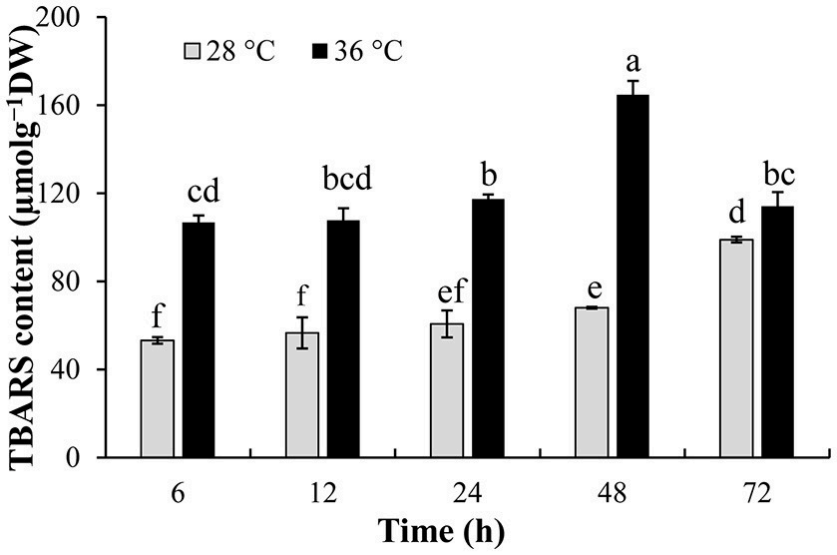
Effect of high temperature on TBARS content. *P. ostreatus* mycelia were first incubated at 28°C, then mycelia were treated with 28 and 36°C for 2 days, respectively. Data were analyzed by Duncan's ANOVA test. Error bars represent the standard deviation of three replicates. Different letters indicate significant differences between the columns (*P* ≤ 0.05 according to Duncan's multiple range test).

TBARS content of *P. ostreatus* mycelia was measured to detect the oxidative damage of cell membranes caused by high temperature. TBARS content increased significantly after *P. ostreatus* mycelia were incubated at 36°C for 6–72 h, and was significantly (*p* ≤ 0.05) higher than that incubated at 28°C (53.14 μmolg^−1^DW) (Figure 2). *P. ostreatus* cultured at 36°C for 48 h had the greatest oxidative damage to cell membranes, and TBARS content was as high as 164.69 μmolg^−1^DW, 142.3% higher than that incubated at 28°C for 48 h.

### Multivariate analysis of GC/MS data

**Figure 3:**
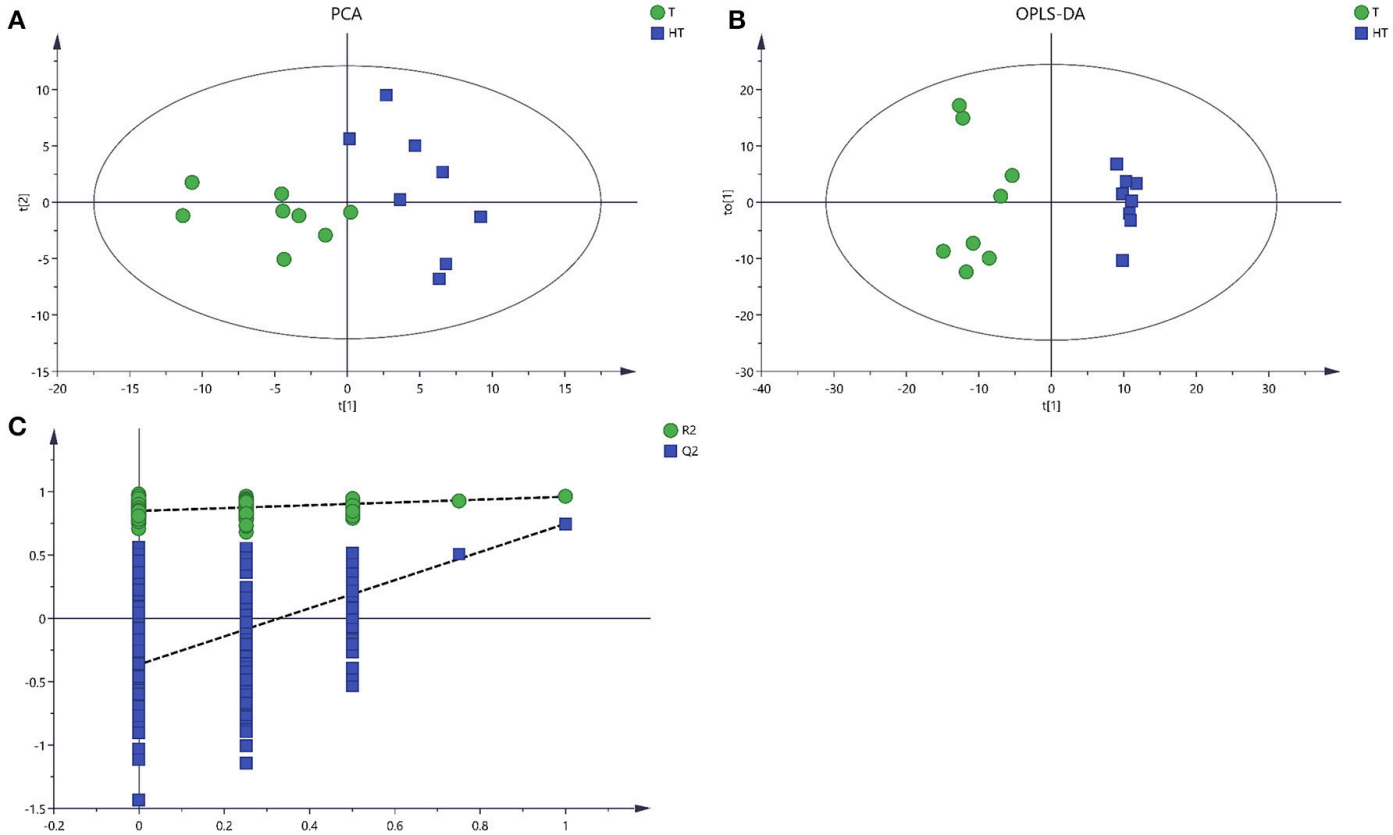
Score plots for extracellular metabolites of GC/MS data. **(A)** The score plots of PCA for metabolites profiles. **(B)** OPLS-DA score plots from metabolites profiles. **(C)** A 200 times permutation test of OPLS-DA models. Green sample T was collected from 28°C. Blue sample HT was collected from which was treated with 36°C.

There were 143 metabolites from 28° and 36°C samples putatively identified by GC/MS with good reproducibility. To analyze the metabolite profiles, an unsupervised PCA was first applied to the dataset (Figure 3A). The unsupervised PCA model with three components was constructed according to the parameters in Table 1. PCA showed an obvious and regular variation of different samples which were treated with different temperatures. This plot suggested that high temperature could significantly change the concentration of extracellular metabolites. To further confirm the overall differences of metabolic profile associated with high temperature, a supervised OPLS-DA model was constructed with the parameters in Table 1. The plot scores showed a clearer separation, with metabolites collected from 28°C fully in the left half and those treated with 36°C fully in the right half (Figure 3B). A 200-time permutation test showed the OPLS-DA model was not over fitting (Figure 3C). The Y-axis intercepts were: R2 (0.0, 0.847), Q2 (0.0, −0.365).

**Table 1:** Statistical data of different models obtained from GC/MS analyses.

**Model**	**R^2^X(cum)**	**R^2^Y(cum)**	**Q^2^(cum)**	**R2**	**Q2**
PCA-X	0.504		0.12		
OPLS-DA	0.289	0.959	0.745	0.847	−0.365

### Identification of differential metabolites from GC/MS data

High temperature can cause changes in metabolic pathways of multiple metabolites, thereby changing the extracellular metabolite content and concentration. The differential metabolites were selected based on the VIP values larger than 1.0 (VIP > 1) and *p* values less than 0.05 (*p* < 0.05). Based on these criteria, a total of 36 differential metabolites were selected from the extracellular fluid after high temperature, including 10 organic acids, 5 amines, 3 saccharides, 4 amino acids, 3 alcohols, 3 esters and 8 other metabolites (Table 2). The results for each metabolite indicating increase or decrease in comparison of 36° vs. 28°C is shown in Table 2. Among the 36 metabolites, most show an increase (I) in concentration, and only 9 showed decreases (D). This indicated that high temperature can inhibit the synthesis of some metabolites. In addition, some metabolites were detected only in the extracellular fluid after high temperature stress, including tartaric acid, itaconic acid, 2-amino-3-(4-hydroxyphenyl) propanoic acid, 6-hydroxy caproic acid and 9-phenanthrol.

**Table 2:** Differential metabolites response to high temperature from GC/MS analyses.

**Names**	**Metabolites**	**Change**	**Names**	**Metabolites**	**Change**
Organic acids	Tartaric acid	I^*^	Amino acids	L-Valine	I
	Itaconic acid	I^*^		L-Tyrosine	I
	2-amino-3-(4-hydroxyphenyl) propanoic acid	I^*^		N-Acetyl-L-tryptophan	D
	6-hydroxy caproic acid	I^*^		Canavanine	D
	6-Hydroxynicotinic acid	I	Alcohols	Allo-inositol	I
	D-Glyceric acid	I		20 alpha-Hydroxycholesterol	I
	Pelargonic acid	I		Phytosphingosine	D
	Acetylsalicylic Acid	I	Esters	Mono (2-ethylhexyl) phthalate	I
	Gluconic acid	I		D-erythronolactone	I
	Oxalic acid	I		4-hydroxybutyrate	D
Amines	Malonamide	I	Others	9-Phenanthrol	I^*^
	Spermidine	I		Cyclohexane-1,2-diol	I
	N-Acetyl-D-galactosamine	I		5,6-Dimethylbenzimidazole	I
	5-Methoxytryptamine	D		22-Ketocholesterol	I
	Alpha-D-glucosamine 1-phosphate	D		Butyraldehyde	D
Carbohydrates	Trehalose	I		4-Vinylphenol dimer	D
	Fucose	I		Phosphate	I
	Lyxose	I		Carnitine	D

* *It was detected only in the extracellular fluid after high temperature stress*.

### Multivariate analysis of LC/MS data

**Figure 4:**
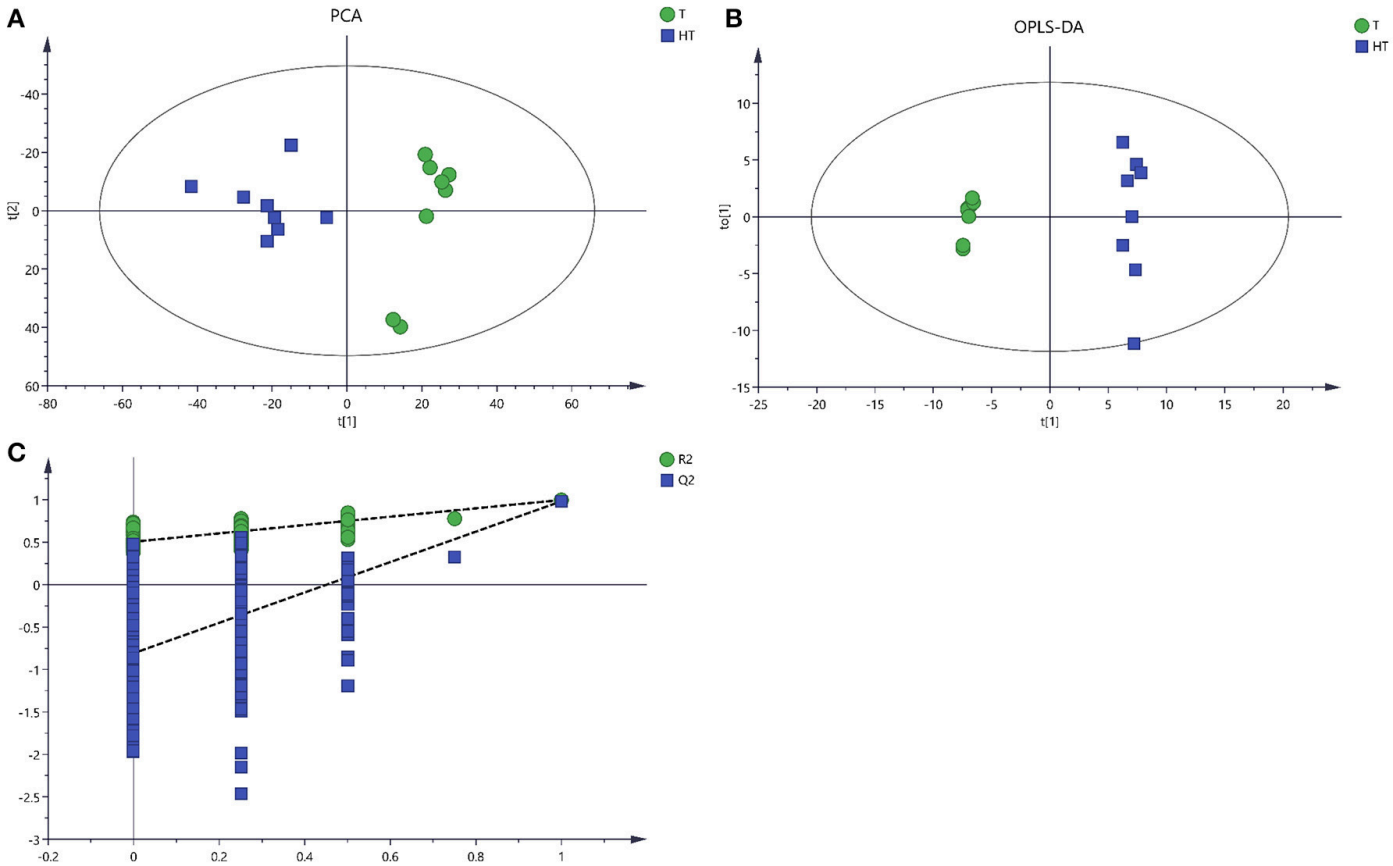
Score plots for extracellular metabolites of LC/MS data. **(A)** The score plots of PCA for metabolites profiles. **(B)** OPLS-DA score plots from metabolites profiles. **(C)** A 200 times permutation analysis were used to validated OPLS-DA models. Green sample T was collected from 28°C. Blue sample HT was collected from which was treated with 36°C.

After processing with database, there were 1449 metabolites from 28° and 36°C samples putatively identified by LC/MS. The unsupervised PCA and the supervised OPLS-DA were applied to analysis the total variation and distinguish the overall differences of the metabolic profile. PCA and OPLS-DA provided clear variation of metabolites, and there was significant separation from samples treated with 28° and 36°C (Figures 4A,B). The PCA model and OPLSA-DA model were constructed according to the parameters in Table 3.

**Table 3:** Statistical data of different models obtained from LC/MS analyses.

**Model**	**R^2^X(cum)**	**R^2^Y(cum)**	**Q^2^(cum)**	**R2**	**Q2**
PCA	0.589		0.432		
OPLS-DA	0.844	0.996	0.981	0.504	−0.808

### Identification of differential metabolites from LC/MS data

Based on the VIP values larger than 1.0 (VIP > 1) and *p* values less than 0.05 (*p* < 0.05), 105 differential metabolites in response to high temperature were selected using LC/MS, including 35 organic acids, 7 amines, 9 glycosides, 4 esters, 4 saccharides, 5 alkalis, 11 amino acids and 30 other metabolites (Table 4). Among them, 88.57% show an increase (I) in concentration, and only 11.43% showed decreases (D). Some were at low levels in the extracellular fluid collected from 28°C, whereas their content was greater after 36°C incubation, including 11E,13-tetradecadienal, 2-amino-2-norbornanecarboxylic acid, feruloylputrescine, beta-kamlolenic acid, diphenylpyraline and 4-oxoproline.

**Table 4:** Differential metabolites response to high temperature from LC/MS analyses.

**Names**	**Metabolites**	**Change**	**Names**	**Metabolites**	**Change**	**Names**	**Metabolites**	**Change**
Organic acids	2-Amino-2-Norbornanecarboxylic acid	I^*^	Amines	Feruloylputrescine	I^*^	Amino acids	N-Alpha-acetyllysine	I
	Beta-kamlolenic acid	I^*^		N-Acetyl-D-Galactosamine 6-phosphate	I		L-Valine	I
	9,12,13-trihydroxy-10-octadecenoic acid	I		2-Naphthylamine	I		N, N-Dihydroxy-L-tryptophan	I
	N-(6-aminohexanoyl)-6-aminohexanoic acid	I		Tryptamine	I		4-Hydroxy-L-threonine	I
	13,14-dihydro-15-keto-PGD2	I		L-Glutamine	I		L-Arginine	I
	2-Ketohexanoic acid	I		Spermidine	I	Others	11E,13-Tetradecadienal	I^*^
	5-Aminoimidazole ribonucleotide	I		N-Gluconyl ethanolamine phosphate	I		Diphenylpyraline	I^*^
	alpha-kamlolenic acid	I	Glycosides	5′-Deoxy-5′-(methylthio) adenosine	I		Amaroswerin	I
	6-Acetamido-3-aminohexanoate	I		Guanosine	I		Butethal	I
	4-Aminohippurate	I		Deoxyguanosine	I		Propylthiouracil glucuronide	I
	Loxoprofen	I		Isoguanosine	I		10-Acetyl-3,7-dihydroxyphenoxazine	I
	(9R,13R)-1a,1b-dihomo-jasmonic acid	I		Uridine	I		4-Methylphenyl acetone	I
	Ketoleucine	I		5′-CMP	I		Enoximone sulfone	I
	6E,8E,12E,14E-Hexadecatetraen-10-ynoic acid	I		8-Oxo-dGMP	I		deoxyguanosine 5′-monophosphate	I
	Oxoglutaric acid	I		Palmitoyl glucuronide	I		Glutathione, oxidized	I
	3-Methylsuberic acid	I		Meptazinol glucuronide	D		Luteolinidin	I
	Gluconic acid	I	Esters	N-3-oxo-tetradec-7(Z)-enoyl-L-Homoserine lactone	I		2-Hexylbenzothiazole	I
	ketoisovaleric acid	I		3-Hydroxy-4-butanolide	I		Catechin 5-O-beta-D-apiofuranoside	I
	6-Hydroxynicotinic acid	I		Methyldopate	I		Leptophylloside	I
	N-Acetyl-L-glutamic acid	I		cis-Acetylacrylate	I		Khellin	I
	1-Naphthyl-β-D-glucuronide	I	Carbohydrates	Trehalose	I		Penaresidin A	I
	11-deoxy-11-methylene-15-keto-PGD2	I		2-Deoxy-D-Ribose	I		3,8-dimethyldec-7-en-1-yl trihydrogen diphosphate	I
	3-Dehydroquinic acid	I		L-Erythrulose	I		11-deoxy-11-methylene-15-keto-PGD2	I
	3′-Deoxydryopteric acid	I		D-glycero-D-manno-heptose 7-phosphate	D		2-Undecyl-4(1H)-quinolinone N-oxide	I
	fumarylacetic acid	I	Alkalines	Tigloidine	I		(-)-Medicarpin	I
	cis-2-Methylaconitate	I		Samandarine	I		Chlorate	I
	(S)-dihydrolipoic acid	I		Glycerophosphocholine	I		Gingerenone A	I
	Glucoheptonic acid	I		Narciclasine	I		Phosphate	I
	Threonic acid	I		Kifunensine	I		L-Xylonate	I
	4-(n-nonyl) Benzeneboronic acid	I	Amino acids	L-Leucine	I		Erinapyrone C	D
	2-Hydroxyadipic acid	I		L-Phenylalanine	I		Diphenyl disulfide	D
	2-Oxo-4E-hexenoic acid	D		L-Arginine	I		3,4-Dehydrothiomorpholine-3-carboxylate	D
	3,4-Dehydrothiomorpholine-3-carboxylate	D		4-Oxoproline	I^*^		7-N, N-Dimethylamino-1,2,3,4,5-pentathiocyclooctane	D
	Salicyl acyl glucuronide	D		L-Tyrosine	I		Ribose-1-arsenate	D
	Chorismic acid	D		Histidylleucine	I		Zinc acetate	D

* *It was at low levels in the extracellular fluid collected from 28°C, whereas their content was greater after 36°C incubation*.

### Effect of some exogenous metabolites on mycelial growth and conidial germination of *T. asperellum in vitro*

Some exogenous metabolites which the content increased after 36°C for 2 days were used to determine the effects on *T. asperellum* mycelial growth. Different concentration of exogenous metabolites exhibited different effects on the mycelial growth of *T. asperellum*. Except kifunensine, all applied exogenous metabolites promoted the mycelial growth at any given concentration (50–250 μM) (Table 5). Kifunensine had an inhibition effect on mycelial growth at a concentration of 250 μM. With the increase of concentration, almost all metabolites showed the promotion tendency and significant stimulation effects on mycelial growth. The colony diameter of T11 reached its highest at the concentration of 250 μM, and the promotion effects under 250 μM were significantly (*p* ≤ 0.05) higher than those at other concentrations (50–100 μM). On the whole, these exogenous metabolites had stimulation effects on mycelial growth.

**Table 5:** Effect of exogenous metabolites on the growth of *T. asperellum* T11 mycelia.

**Names**	**Exogenous metabolites**	**Colony diameters of** ***T. asperellum*** **(cm)**			
		**0 μM**	**50 μM**	**100 μM**	**250 μM**
Organic acids	Salicylic acid	5.87 ± 0.04 d	6.43 ± 0.05 c	6.87 ± 0.09 b	7.20 ± 0.08 a
	6-Hydroxynicotinic acid	5.87 ± 0.04 d	6.57 ± 0.09 c	6.87 ± 0.09 b	7.17 ± 0.11 a
	Gluconic acid	5.87 ± 0.04 d	6.67 ± 0.04 c	6.97 ± 0.11 b	7.37 ± 0.05 a
Amino acids	L-Tyrosine	5.87 ± 0.04 c	6.87 ± 0.09 b	6.97 ± 0.09 b	7.20 ± 0.08 a
	L-Valine	5.87 ± 0.04 c	6.57 ± 0.09 b	6.67 ± 0.04 b	6.97 ± 0.11 a
	L-Leucine	5.87 ± 0.04 d	6.43 ± 0.05 c	6.67 ± 0.04 b	7.07 ± 0.26 a
Amines	Spermidine	5.87 ± 0.04 d	6.17 ± 0.11 c	6.43 ± 0.05 b	6.67 ± 0.04 a
	L-Glutamine	5.87 ± 0.04 d	6.37 ± 0.04 c	6.57 ± 0.09 b	7.00 ± 0.08 a
	Malonamide	5.87 ± 0.04 d	6.23 ± 0.09 c	6.43 ± 0.05 b	6.87 ± 0.09 a
Carbohydrates	Trehalose	5.87 ± 0.04 c	7.00 ± 0.08 b	7.13 ± 0.11 b	7.60 ± 0.08 a
	Fucose	5.87 ± 0.04 d	6.87 ± 0.09 c	7.17 ± 0.11 b	7.33 ± 0.04 a
	L-Erythrulose	5.87 ± 0.04 c	6.67 ± 0.04 b	6.87 ± 0.09 a	7.00 ± 0.08 a
Glycosides	Guanosine	5.87 ± 0.04 d	6.43 ± 0.05 c	6.67 ± 0.04 b	6.97 ± 0.11 a
	Uridine	5.87 ± 0.04 d	6.37 ± 0.04 c	6.67 ± 0.09 b	6.87 ± 0.09 a
	deoxyguanosine	5.87 ± 0.04 d	6.50 ± 0.08 c	6.87 ± 0.09 b	7.07 ± 0.26 a
Esters	3-(2-Ethylhexyl) phthalate	5.87 ± 0.04 d	6.17 ± 0.11 c	6.43 ± 0.05 b	6.80 ± 0.08 a
	d-erythronolactone	5.87 ± 0.04 d	6.20 ± 0.11 c	6.43 ± 0.05 b	6.87 ± 0.09 a
	N-3-oxo-tetradec-7(Z)-enoyl-L-Homoserine lactone	5.87 ± 0.04 d	6.20 ± 0.08 c	6.40 ± 0.08 b	6.90 ± 0.07 a
Alkalines	Glycerophosphocholine	5.87 ± 0.04 d	6.17 ± 0.11 c	6.57 ± 0.09 b	6.90 ± 0.07 a
	Narciclasine	5.87 ± 0.04 d	6.17 ± 0.09 c	6.47 ± 0.05 b	6.80 ± 0.08 a
	Kifunensine	5.87 ± 0.04 a	5.80 ± 0.08 a	5.83 ± 0.08 a	5.47 ± 0.05 b
Others	Glutathione, oxidized	5.87 ± 0.04 d	6.20 ± 0.08 c	6.57 ± 0.09 b	6.90 ± 0.05 a
	diphenylpyraline	5.87 ± 0.04 c	6.00 ± 0.08 bc	6.17 ± 0.08 ab	6.27 ± 0.04 a
	butethal	5.87 ± 0.04 d	6.17 ± 0.11 c	6.47 ± 0.05 b	6.87 ± 0.09 a

*Values with a different letter within the same line are significantly different at P ≤ 0.05 according to Duncan's test. Numbers followed by “±” are the standard errors (SEs)*.

Similarly, these metabolites were also used to determine the effects on T11 conidial germination. Compared with their effects on mycelial growth, these exogenous metabolites had similar promotion effects on conidial germination (Table 6). Kifunensine was also unsuitable for conidial germination either in the given concentration, and high concentration (250 μM) of kifunensine could significantly (*p* ≤ 0.05) inhibited the conidial germination. Moreover, there were no significant promotion effects on conidial germination at any given concentration of diphenylpyraline. For other metabolites, there was a stimulation tendency on conidial germination accompanied by an increase in the concentration of exogenous metabolites and significant (*p* ≤ 0.05) promotion effects were found at concentrations of 100 and 250 μM.

**Table 6:** Effect of exogenous metabolites on *T. asperellum* T11 conidial germination.

**Names**	**Exogenous metabolites**	**Germination rate of** ***T. asperellum*** **conidia (%)**			
		**0 μM**	**50 μM**	**100 μM**	**250 μM**
Organic acids	Itaconic acid	30 ± 0.82 d	45 ± 4.32 c	63 ± 6.48 b	78 ± 1.41 a
	6-Hydroxynicotinic acid	30 ± 0.82 d	36 ± 2.16 c	46 ± 2.94 b	70 ± 1.41 a
	Gluconic acid	30 ± 0.82 d	42 ± 1.41 c	53 ± 2.94 b	65 ± 2.16 a
Amino acids	L-Tyrosine	30 ± 0.82 d	34 ± 2.16 c	45 ± 2.16 b	57 ± 3.74 a
	L-Valine	30 ± 0.82 d	38 ± 0.82 c	48 ± 2.16 b	70 ± 2.94 a
	L-Leucine	30 ± 0.82 d	34 ± 1.41 c	46 ± 3.55 b	59 ± 2.16 a
Amines	Spermidine	30 ± 0.82 d	36 ± 1.69 c	48 ± 2.94 b	65 ± 1.41 a
	L-Glutamine	30 ± 0.82 d	38 ± 1.63 c	52 ± 2.16 b	74 ± 3.74 a
	Malonamide	30 ± 0.82 d	34 ± 1.41 c	46 ± 2.16 b	58 ± 2.16 a
Carbohydrates	Trehalose	30 ± 0.82 d	36 ± 1.41 c	53 ± 3.55 b	75 ± 2.94 a
	Fucose	30 ± 0.82 d	41 ± 3.74 c	54 ± 2.16 b	68 ± 2.94 a
	L-Erythrulose	30 ± 0.82 d	39 ± 2.94 c	46 ± 1.41 b	57 ± 3.55 a
Glycosides	Guanosine	30 ± 0.82 d	42 ± 2.49 c	58 ± 4.24 b	76 ± 3.74 a
	Uridine	30 ± 0.82 d	35 ± 1.41 c	44 ± 2.49 b	56 ± 3.55 a
	Deoxyguanosine	30 ± 0.82 d	36 ± 2.16 c	50 ± 3.55 b	65 ± 4.54 a
Esters	3-(2-Ethylhexyl) phthalate	30 ± 0.82 d	34 ± 1.41 c	45 ± 2.94 b	56 ± 2.16 a
	D-erythronolactone	30 ± 0.82 d	38 ± 3.74 c	56 ± 1.41 b	65 ± 3.74 a
	N-3-oxo-tetradec-7(Z)-enoyl-L-Homoserine lactone	30 ± 0.82 d	32 ± 2.16 c	38 ± 0.82 b	43 ± 1.41 a
Alkalines	Glycerophosphocholine	30 ± 0.82 d	33 ± 1.41 c	36 ± 1.41 b	46 ± 3.74 a
	Narciclasine	30 ± 0.82 d	45 ± 4.32 c	58 ± 2.94 b	76 ± 4.54 a
	Kifunensine	30 ± 0.82 a	30 ± 1.41 a	29 ± 1.41 a	25 ± 0.82 b
Others	Glutathione, oxidized	30 ± 0.82 d	35 ± 2.16 c	43 ± 3.74 b	51 ± 3.55 a
	Diphenylpyraline	30 ± 0.82 b	31 ± 1.41 a	32 ± 0.82 a	31 ± 1.41 a
	Butethal	30 ± 0.82 d	32 ± 0.82 c	38 ± 2.16 b	43 ± 2.94 a

*Values with a different letter within the same line are significantly different at P ≤ 0.05 according to Duncan's test. Numbers followed by “±” are the standard errors (SEs)*.

**Figure 5:**
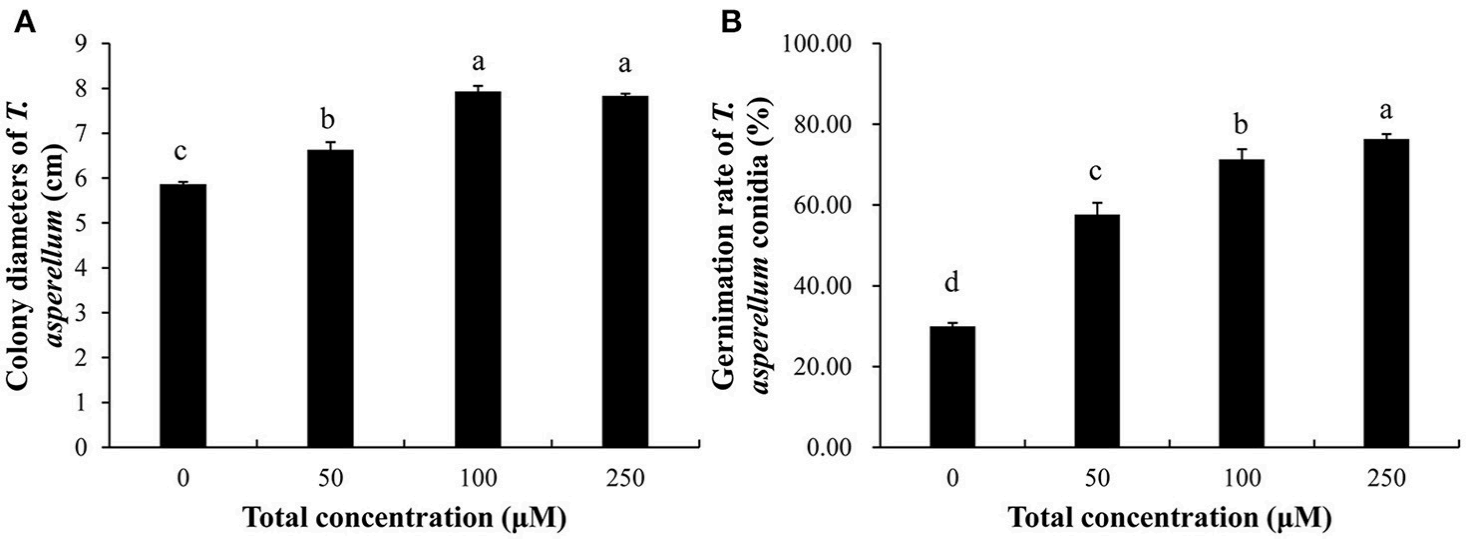
Effect of combination of exogenous metabolites on the growth of *T. asperellum*. **(A)** Promoting effect on mycelial growth. **(B)** Promoting effect on conidial germination. Data were analyzed by Duncan's ANOVA test. Error bars represent the standard deviation of three replicates. Different letters indicate significant differences between the columns (*P* ≤ 0.05 according to Duncan's multiple range test).

The medium which contained all these metabolites at concentrations ranging from 50 to 250 μM exhibited significant (*p* ≤ 0.05) stimulating effects on mycelial growth and conidial germination. The T11 colony diameter was highest (7.93 cm) at 100 μM and the promotion effects under 100 and 250 μM were significantly (*p* ≤ 0.05) higher than other concentrations (0, 50 μM) (Figure 5A). Moreover, there was a tendency of an increase in conidial germination with the increasing of metabolites concentrations (50–250 μM) and conidial germination rate in the presence of metabolites was significantly (*p* ≤ 0.05) higher than that on water agar petri dishes (Figure 5B). The maximum conidial germination rate was 76.33% on the plates with metabolites at 250 μM.

## Discussion

In China, *P. ostreatus* is mainly cultivated in a traditional way in vinyl houses. This can cause it to be easily affected by the external environmental temperature during spawn running period. The mycelial growth rate and the high yield of fruiting bodies are closely related to the appropriate temperature conditions (Zervakis et al., 2001). For the Chinese traditional agricultural cultivation way, high temperature is one of the common abiotic stresses. If the cultivation houses are not ventilated in time, the internal temperature of cultivation bags will rise, resulting in the occurrence of spawn-burning and often the green mold disease (Zhang et al., 2016).

Temperature is an important abiotic factor that affects intracellular metabolism and modulates fungal growth (Medina et al., 2015). In this study, we collected extracellular cultures of *P. ostreatus* at different temperatures and compared their effects on the growth speed and conidia germination rate of *T. asperellum*. The results showed that extracellular fluid collected from which subjected to high temperature stresses for 2 days (32°, 36°, and 40°C) exhibited similar promoting effects on T11 mycelial growth and conidial germination (Figure 1). It indicated that high temperature changed the extracellular metabolites of *P. ostreatus*, which provided a good basis for mycelial growth and conidial germination of *T. asperellum*. Cell membrane has an important physiological function, which makes cells maintain stable intracellular environment and can adjust or select substances into and out of cells (Sant et al., 2016). With the increase of temperature, the levels of membrane lipid peroxidation will be increased (Kong W. W. et al., 2012). For *P. ostreatus* cultivation, 36°C is a common high temperature in the summer of China and it can significantly inhibit the mycelial growth (Oei, 1991). *Pleurotus ostreatus* mycelia subjected to high temperature (36°C) produced higher TBARS content than that cultured at 28°C, and the TBARS content reached the maximum at 36°C for 48 h (Figure 2). The increase of TBARS content will lead to enhanced membrane permeability and exosmosis of electrolytes (Zhu et al., 2009; Guyot et al., 2015). These results indicated that *P. ostreatus* mycelia suffered greater damage on membrane lipid after high temperature (36°C) and *P. ostreatus* mycelia treated with 36°C for 48 h was a suitable treatment for studying changes in extracellular metabolites.

Enhanced temperature will accelerate the accumulation of ROS which is an important signal, and can activate multiple metabolic pathways in the cell to alleviate the damage to cells caused by environmental temperature (Kumar et al., 2011). With the increase of cell membrane permeability, more intracellular metabolites permeated into the extracellular region. Metabolomics provides an accurate approach to study the response of these metabolites to living conditions and environmental factors (Zhang et al., 2010). The PCA, an unsupervised method could clearly confirm the intrinsic variation of metabolites and the stability of the whole analysis process (Brusco, 2014). To more precisely analyze the overall differences in metabolic profiles between groups, a supervised method (OPLS-DA) was practical (Song et al., 2013). In this study, application of multivariate PCA and OPLS-DA on the quantified metabolites revealed clear separation and clustering in metabolites of sample T and HT (Figures 3, 4). These results indicated that there is changes in composition and concentration of extracellular metabolites of *P. ostreatus* caused by high temperature.

GC/MS and LC/MS are methods to detect molecules with different chemical characteristics (Doerfler et al., 2013). In this study, a total of 36 different metabolites were identified by GC/MS which mainly included seven kinds of metabolites, such as, organic acids, amines, carbohydrates, amino acids, alcohols, esters and some other metabolites (Table 2). In contrast with GC/MS, there were more metabolites identified by LC/MS. A total of 105 differential metabolites were identified by LC/MS (Table 4). There were also six compounds detected simultaneously by GC/MS and LC/MS, and the changing tendency of the concentration was consistent. These six compounds were valine, tyrosine, gluconic acid, trehalose, spermidine, and 6-hydroxynicotinic acid. It indicated that GC/MS and LC/MS can detect the change of metabolite accurately, and integrated GC/MS and LC/MS was helpful to comprehensively understand the changes of metabolites (Lozano et al., 2012).

A total of 141 differential metabolites were detected by using these two methods simultaneously. After high temperature treatment, 84.4% of extracellular metabolites increased in the content while 15.6% of them decreased. Several amino acids increased after high temperature, including L-valine, L-tyrosine, L-leucine, L-phenylalanine, 4-oxoproline, etc. Elevated valine, phenylalanine and tyrosine levels help maintain cell wall stability at high temperature and enhance stress tolerance (Cassab, 1998; Suguiyama et al., 2014). High temperature results in an increase in intracellular trehalose to alleviate oxidative damage, and thereby increasing resistance to heat stress (Trevisol et al., 2011). However, some nucleoside analogs which rarely permeate outside the cell were also detected by LC/MS. For example, uridine and guanosine which belong to growth factor are the standard nucleosides which make up nucleic acids. These results might indicate that high temperature increases the permeability of the cell membrane or damages the cell membrane, causing leakage of intracellular nutrients and substances which can provide nourishment for the colonization of *Trichoderma* spp.

In the process of *Trichoderma* infecting other fungi, competition for nutrients is a common mechanism for *Trichoderma* to inhibit the growth of other fungi. *Trichoderma* would grow rapidly by using exogenous nutrients to occupy the niche (Benítez et al., 2004; Segarra et al., 2010). In this study, some randomly selected exogenous metabolites whose content increased after high temperature showed similar stimulatory effects on *T. asperellum* mycelial growth and conidial germination (Tables 5, 6). There are many primary metabolites which are essential for life activities, such as organic acids, amino acids, esters and carbohydrates. Except kifunensine, almost all of the metabolites we selected had higher promotion effects with increasing concentrations (50–250 μM). Kifunensine which originally isolated from *Kitasatosporia kifunense* is a potent inhibitor of mannosidase I enzyme (Males et al., 2017). Kifunensine may serve as a new substance for the control of *Trichoderma*. Previous studies had also found that some substances could inhibit *Trichoderma* (Lee et al., 2017; Mwangi et al., 2017). These results may shed light on control of green mold disease. Under the treatment of the combination of all these selected metabolites in the given concentrations (50–250 μM), *T. asperellum* was found to have a significantly faster growth speed and higher conidial germination (Figure 5). High temperature increased the content of these extracellular metabolites, thus further provided a good condition for the growth of *T. asperellum*. These results indicated that more abundant extracellular nutrients of *P. ostreatus* after high temperature treatment provided a good basis for the large-scale growth of *T. asperellum*.

This is the first study of stimulatory effects of extracellular metabolites of *P. ostreatus* collected from high temperature treatments on *T. asperellum* mycelial growth and conidial germination. GC/MS in combination with LC/MS profiled more comprehensive extracellular different metabolites of *P. ostreatus* in response to high temperature. These extracellular metabolites continuously promoted the mycelial growth and conidial germination of *T. asperellum* as these metabolites increased in concentrations. Taken together, extracellular metabolites of *P. ostreatus* after high temperature treatment are beneficial to the growth of *T. asperellum*, which may provide a new viewpoint for the outbreak of green mold disease during the cultivation of *P. ostreatus*.

## Author contributions

ZQ: conceived, designed and performed the experiments, analyzed the data, wrote and revised the manuscript; JZ and CH: conceived and designed the experiments; XW and CH: revised the manuscript.

### Conflict of interest statement

The authors declare that the research was conducted in the absence of any commercial or financial relationships that could be construed as a potential conflict of interest.
